# Non-Sleep Deep Rest Relaxation and Virtual Reality Therapy for Psychological Outcomes in Patients with Coronary Artery Disease: A Pilot Randomized Controlled Trial

**DOI:** 10.3390/jcm13237178

**Published:** 2024-11-26

**Authors:** Adam Wrzeciono, Błażej Cieślik, Pawel Kiper, Joanna Szczepańska-Gieracha, Robert Gajda

**Affiliations:** 1 Faculty of Physiotherapy, Wroclaw University of Health and Sport Sciences, 51-612 Wroclaw, Poland; 2 Healthcare Innovation Technology Lab, IRCCS San Camillo Hospital, 30126 Venice, Italy; 3 Gajda-Med District Hospital, 06-100 Pułtusk, Poland; 4 Institute of Physical Culture Sciences, Jan Dlugosz University in Częstochowa, 42-200 Częstochowa, Poland

**Keywords:** anxiety, cardiac rehabilitation, depression, psychological intervention, stress

## Abstract

**Background:** While cardiac rehabilitation (CR) primarily focuses on restoring physical strength, preventing relapse, and reducing rehospitalization rates, psychological interventions play a complementary role by supporting mental health, which is crucial for patients’ long-term adherence and overall recovery. The effectiveness of psychological interventions in CR is debated, and while technologies like virtual reality (VR) therapy show promise, they have limitations for patients with coronary artery disease (CAD). Therefore, this study examines non-sleep deep rest (NSDR) relaxation, a novel and easily implementable technique, and compares its impact on depression, anxiety, and stress with VR therapy and standard care. **Methods:** Forty-five CAD patients undergoing CR in ambulatory conditions were divided into three groups: the NSDR group, which received eight sessions of NSDR relaxation as part of their rehabilitation; the VR group, which received eight sessions of VR therapy as part of their rehabilitation; and the control group, which received standard care including Schultz Autogenic Training (SAT). The outcomes were measured using the Hospital Anxiety and Depression Scale (HADS) and the Perception of Stress Questionnaire (PSQ). **Results:** Both NSDR relaxation and VR therapy were effective in reducing the HADS total score, anxiety levels, the PSQ general score, and emotional tension. No significant differences were observed between the two treatment approaches. However, SAT was found to be insufficient for effectively improving the mental state of cardiac patients. **Conclusions:** This study suggests that NSDR relaxation is an effective psychotherapeutic intervention in CR. NSDR and VR therapy showed similar benefits, offering promising alternatives to traditional methods. Integrating these techniques could enhance patient outcomes and adherence in CR. Further research is needed to refine these interventions and optimize their clinical application.

## 1. Introduction

Cardiac rehabilitation (CR) is a comprehensive program designed for patients recovering from cardiovascular events. This multifaceted approach aims to restore physical functioning, reduce the risk of recurrent cardiovascular events, and decrease the likelihood of rehospitalization. Traditionally, CR focuses on physical recovery through supervised exercise, lifestyle modification, and medical management, aiming to enhance cardiovascular health and prevent future complications [1]. Given the psychological and emotional challenges associated with cardiovascular disease (CVD) [2], including coronary artery disease (CAD), which remains a leading cause of morbidity and mortality worldwide [3], recent studies increasingly emphasize the critical role of psychological support in CR, as mental health significantly influences patients’ adherence to lifestyle changes and their engagement in the rehabilitation process [4].

Exercise-based CR has been shown to improve psychological well-being, with a 12-month CR program significantly reducing clinical psychological distress [5]. Another study found that an early 3-week CR program for patients who had undergone coronary artery bypass grafting effectively reduced state anxiety in patients with mild anxiety-depressive symptoms but was less effective for those with severe symptoms [6]. The literature widely documents the association between psychological factors, such as symptoms of depression and anxiety, along with high stress levels, and an increased risk of CVD [7,8,9]. Moreover, depression and anxiety seem to be associated with poor physical performance in CR outpatients, particularly resulting in low exercise tolerance among patients with CAD [9]. In response to the complex interaction between CVD and psychological disorders, standard CR programs often include a psychological treatment component [2,10]. However, the effectiveness of specific psychological interventions within CR remains a topic of debate.

A meta-analysis by Albus et al. revealed that the benefits of specific psychological interventions for CR patients could not be generalized; therefore, these interventions should be tailored to the patients and conditions where they are most effective [11]. In our recent meta-analysis, we obtained similar results regarding the limited effectiveness of psychologically enhanced CR in reducing depression and anxiety levels [12]. Due to these limitations, methods are being sought to increase the effectiveness of CR in mental health.

In recent years, the treatment of anxiety and depression symptoms has increasingly benefited from modern technologies, including virtual environments [13,14]. Our previous research indicated that integrating virtual reality (VR) therapy into standard rehabilitation programs had a positive impact on patients with coronary artery disease. However, several limitations have been identified that restrict the use of this technology among patients with CAD [15]. Therefore, this study evaluates the effectiveness of therapeutic recording within non-sleep deep rest (NSDR) concept. NDSR is a relatively novel technique that shares certain similarities with other established relaxation techniques. Its distinctive combination of guided prompts and emphasis on maintaining consciousness may set it apart as a novel approach to relaxation. Moreover, this technique does not require special training or expertise, making it a feasible option for implementation in various settings [16].

To our knowledge, no studies have evaluated NSDR as a psychological component of comprehensive CR. Therefore, the main purpose of this study was to assess the impact of therapeutic recording within the NSDR concept (NSDR relaxation) on reducing depression and anxiety symptoms, as well as perceived stress levels, in a group of patients with CAD participating in the second phase of CR in ambulatory conditions. Furthermore, this study aimed to compare the effectiveness of NSDR relaxation with that of VR therapy and standard care.

## 2. Materials and Methods

### 2.1. Study Design and Setting

The study was designed as a parallel-group randomized controlled trial. Participants were recruited from the Cardiology Center “Pro Corde” in Wroclaw, Poland, a cardiac clinic that provided the second stage of CR in ambulatory conditions. They were allocated into three equal groups (1:1:1 ratio) using the block randomization method. The randomization was supervised by an independent researcher and remained confidential until all participants were registered and assigned to their respective groups. The randomization sequence was generated using computer software (www.randomization.com, accessed on 20 October 2024), and participants were enrolled via sequentially numbered, sealed envelopes. The outcomes were evaluated by an outcome assessor who was blinded to the group assignment at two time points: before and after the intervention. Although participants and intervention providers were informed about their group assignments throughout the trial, the planned intervention proceeded without any deviations attributable to its context.

The study protocol was reviewed and approved by the Ethics Committee at Wroclaw University of Health and Sport Sciences, Poland (reference number: 1/2023; date: 24 February 2023). The study was registered in the ClinicalTrials.gov database (NCT06241534). All participants provided written informed consent to participate in the research.

### 2.2. Participants

**Figure 1:**
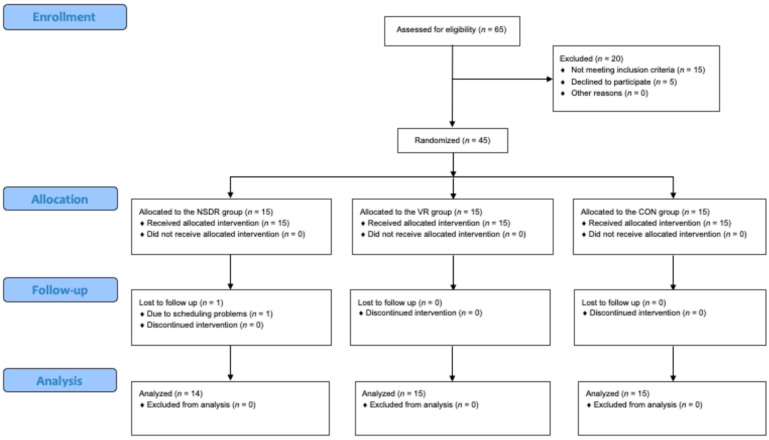
CONSORT study flow diagram. NSDR group: non-sleep deep rest relaxation group; VR group: virtual reality therapy group; CON group: control group.

Following Whitehead et al.’s guidelines for sample size in a pilot randomized trial [17], the study comprised a cohort of 45 patients. As depicted in Figure 1, after an initial eligibility assessment, participants were randomly assigned to one of three treatment groups: NSDR relaxation group (NSDR group), VR therapy group (VR group), and control group (CON group). The inclusion criteria for the study required patients aged between 40 and 85 years, who had been diagnosed with CAD and were actively participating in the second stage of CR. The exclusion criteria included an inability to independently complete the research questionnaires, disturbances in consciousness, psychotic symptoms, or other major psychiatric conditions (either current or documented in medical records), starting psychiatric treatment during the study, contraindications for virtual therapy (such as epilepsy, vertigo, or visual impairments), and refusal to participate at any point during the study.

### 2.3. Interventions

Participants across all three study groups attended eight sessions of standard CR in ambulatory conditions, occurring three times weekly. The rehabilitation protocol included 20 min of interval training on a cycle ergometer and 20 min of general fitness exercises. The intensity of the cycle ergometer interval training was individually determined, taking into account the calculated heart rate reserve for each participant. The general fitness exercises included the use of various fitness equipment, such as a treadmill, pec fly machine, elliptical trainer, rowing machine, and stepper. Throughout the training sessions, cardiac activity was continuously monitored via electrocardiography. The final part of each training session was a calming phase, succeeded by various therapeutic interventions tailored to each group’s assignment.

#### 2.3.1. NSDR Relaxation Group

In addition to their regular treatments, the NDSR group participated in eight 20 min NDSR relaxation. NSDR is a relaxation technique designed to induce a state of deep calm and recovery without actual sleep. It involves listening to a guided script that prompts deep breathing, visualization, and body awareness, allowing individuals to reach a deeply relaxed yet conscious state. Unlike other relaxation techniques, NSDR does not require specialized equipment, extensive training, or post-session recovery, making it a practical alternative to napping or meditation, particularly for those who have difficulty with traditional meditation or daytime sleep [16]. The recording is based on the ‘body scanning technique’, which forms the central component of the therapy. This method seeks to calm the overactive sympathetic part of the autonomic nervous system. Throughout the session, the patient is guided on a ‘journey’ through their body, starting from the feet and moving up to the head. Following the narrator’s instructions, the patient directs their focus to specific areas of the body, attempts to sense those regions, and then relaxes any muscle tension. This relaxation of different muscle groups is integrated with breathing exercises. The narrator directs the patient’s attention to their breath, encouraging deep and mindful breathing, which enhances the relaxation process. Additionally, the recording includes therapeutic suggestions that help the patient choose between opposing states such as overload and relief, holding on and letting go, or acting and sensing. These suggestions also help the patient tap into their inner wisdom, supporting them in making optimal decisions. The session was experienced while seated comfortably in an armchair, wearing high-quality SONY WH-1000XM4 headphones (Sony Corporation, Minato, Tokyo, Japan) equipped with active noise cancelation, in a room designed to ensure tranquility and minimize external disturbances. The therapy was developed by the same therapist who created the VR-based therapy for the other group.

#### 2.3.2. VR Therapy Group

Aside from their regular treatments, the VR group participated in eight 20 min sessions of immersive VR therapy using the VRTierOne device by Stolgraf^®^ (Stanowice, Poland). The therapy aims to alleviate stress and anxiety, enhance mood, trigger positive emotions, and encourage patient engagement in rehabilitation. It was designed around the concept of a Virtual Therapeutic Garden inspired by Ericksonian psychotherapy principles. The immersive experience, involving all senses and employing hypnotic communication, enhanced the effectiveness of VR therapy by fostering patience and perseverance in goal pursuit. The device included VR goggles (HTC VIVE PRO, New Taipei City, Taiwan) capable of showcasing high-resolution images, along with specialized manipulators that translated patient hand gestures into the virtual setting. The visual effects were augmented by ambient sounds such as wind, birdsong, and calm music playing softly in the background to accompany the narrator’s messages. Additional insights into the principles guiding the VRTierOne device can be explored in our previous research [18].

#### 2.3.3. Control Group

As part of the regular CR protocol, the CON group participated in Schultz’s Autogenic Training (SAT). SAT is a relaxation method in which patients, while seated comfortably with their eyes closed, listen to suggestions provided by the therapist. Guided by the therapist’s directions, patients endeavor to breathe slowly and progressively relax different parts of their body. The SAT sessions were administered via CD playback by a psychologist.

### 2.4. Outcomes Measures

The primary outcome measures included two standardized questionnaires: the Hospital Anxiety and Depression Scale (HADS) and the Perception of Stress Questionnaire (PSQ). The initial assessment was conducted for all participants at the start of CR, and the final assessment was carried out after eight sessions of CR.

HADS is a 14-item self-report questionnaire designed to screen for anxiety and depression in patients in non-psychiatric settings. It includes two subscales: HADS-A for anxiety and HADS-D for depression, each consisting of seven items. Both subscales have a cut-off score of 8 out of 21. According to Bjelland et al., the scale has a Cronbach’s α ranging from 0.78 to 0.93 and a test–retest correlation of *r* = 0.80 [19].

The PSQ by Plopa and Makarowski is composed of 27 statements designed to evaluate emotional tension, external stress, intrapsychic stress, and the risk of lying. Patients rate these statements on a five-point Likert scale (true, mostly true, uncertain, mostly not true, not true). The overall score, ranging from 21 to 105 points, determines the patient’s stress level, with higher scores indicating more severe stress symptoms. A cut-off point is set at 60 points. The questionnaire’s reliability, as measured by Cronbach’s alpha, is 0.72 for external stress, 0.81 for emotional tension, and 0.69 for intrapsychic stress [20].

### 2.5. Data Analysis

All statistical analyses were performed using JASP version 0.18.3 (University of Amsterdam, The Netherlands). Categorical variables were reported as frequency counts and percentages, whereas continuous variables were summarized using the mean and standard deviation (SD). The normal distribution of the data was confirmed by the Shapiro–Wilk test. Baseline demographic variables were compared between groups using a one-way analysis of variance (ANOVA) for continuous data and χ^2^ (chi-square) tests for categorical data. To assess the effects of the intervention between groups (pre- vs. post-intervention), a repeated measures ANOVA was conducted, supplemented by Bonferroni correction.

## 3. Results

### 3.1. Participant Characteristic

Out of 65 potential participants, 45 met the inclusion criteria and were randomized for the study. One participant from the NSDR group dropped out due to scheduling problems (Figure 1). Analyses of demographic (Table 1) and clinical (Table 2) data revealed that there were no statistically significant differences between the three study groups at baseline.

### 3.2. Effectiveness of the Interventions

Table 2 presents the mean values and standard deviations (SD). Table 3 depicts the time × group interaction as determined by ANOVA, while Table 4 illustrates the comparison of the effectiveness of the three treatment conditions.

Significant results were found in HADS, HADS-A, PSQ, intrapsychic stress (IS), and external stress (ET). Specifically, for HADS, scores decreased by 30.6% in the NSDR group, 10.2% in the VR group, and increased by 2.0% in the CON group. Analysis of variance revealed a significant time × group interaction for HADS, indicated by an F value of 5.51, effect size η_p_^2^ of 0.21, and a p Value of 0.01. Further analysis exposed a significant difference in effectiveness between the NSDR group and CON group of −4.20 (*p* = 0.03), with no significant difference between the two experimental conditions (*p* < 0.13).

For HADS-A, scores decreased by 28% in the NSDR group, 1.1% in the VR group, and increased by 7.0% in the CON group. Analysis of variance revealed a significant time × group interaction for HADS-A, indicated by an F value of 4.19, effect size η_p_^2^ of 0.17, and a *p* Value of 0.02. Further analysis exposed a significant difference in effectiveness between NSDR group and CON group of −2.47 (*p* < 0.01), with no significant difference between the two experimental condition (*p* < 0.11).

For HADS-D, scores decreased by 33.6% in the NSDR group, 20.4% in the VR group, and by 3.1% in the CON group. Further analyses revealed no significant time × group interaction or between-group differences in effectiveness for HADS-D. For PSQ, scores decreased by 9.7% in the NSDR group, 13.3% in the VR group, and increased by 6.9% in the CON group. Analysis of variance revealed a significant time × group interaction for PSQ, indicated by an F value of 7.27, effect size η_p_^2^ of 0.26, and a *p* Value of <0.01. Further analysis exposed a significant difference in effectiveness between NSDR group and CON group of −9.42 (*p* = 0.02) and between VR group and CON group of −11.60 (*p* < 0.01), with no significant difference between the two experimental conditions. For ES, scores decreased by 2.1% in the NSDR group, 13.4% in the VR group, and increased by 3.7% in the CON group. Further analyses revealed no significant time × group interaction or between-group differences in effectiveness for ES. For IS, scores decreased by 6.9% in the NSDR group, 11.2% in the VR group, and increased by 6.9% in the CON group. Analysis of variance revealed a significant time × group interaction for IS, indicated by an F value of 3.57, effect size η_p_^2^ of 0.15, and a *p* Value of 0.04. Further analysis exposed a significant difference in effectiveness between NSDR group and CON group of −2.47 (*p* < 0.01), with no significant difference between the two experimental condition (*p* < 0.11). Further analysis revealed no significant between-group differences in effectiveness for IS. For emotional tension (ET), scores decreased by 18.4% in the NSDR group, 15.1% in the VR group, and increased by 9.5% in the CON group. Analysis of variance revealed a significant time × group interaction for ET, indicated by an F value of 8.77, effect size η_p_^2^ of 0.30, and a *p* Value of <0.001. Further analysis exposed a significant difference in effectiveness between NSDR group and CON group of −5.91 (*p* < 0.001) and between VR group and CON group of −5.40 (*p* < 0.001), with no significant difference between the two experimental conditions.

## 4. Discussion

Comprehensive CR aims to enhance patients’ physical fitness and mental well-being [21]. Studies indicate the necessity of psychotherapeutic interventions for cardiac patients experiencing heightened symptoms of depression and anxiety [4,22]. On the other hand, it has been observed that SAT, a standard method of psychological support in CR units, is insufficient for effectively improving the mental state of cardiac patients [6,15]. Therefore, the primary aim of this pilot randomized controlled trial was to evaluate the effectiveness of NSDR relaxation in improving psychological outcomes in patients with CAD during the second phase of CR. Our findings provide several insights into the potential benefits of this intervention.

Our study demonstrated that NSDR relaxation could offer significant psychological benefits to cardiac patients. Specifically, the use of NSDR relaxation was associated with reductions in the HADS total score, anxiety levels, the PSQ general score, and emotional tension. These results suggest that NSDR, with its distinctive combination of guided prompts and emphasis on maintaining consciousness, can be an effective alternative to traditional relaxation techniques. This is particularly relevant given the rising interest in mindfulness and meditative practices in contemporary psychological treatment modalities [23,24].

Similarly, VR therapy showed promise in improving psychological well-being among the CAD patients. This is in line with our previous research [15]. The immersive nature of VR therapy likely contributed to its effectiveness, providing an engaging and distracting environment that helped mitigate negative emotional states [25]. This observation aligns with previous research highlighting the potential of VR in treating psychiatric disorders [13]. Specifically, the use of the Virtual Therapeutic Garden concept was associated with reductions in the HADS total score, anxiety levels, the PSQ general score, and emotional tension. This means that both NSDR relaxation and VR therapy demonstrate effectiveness in the same areas. Moreover, there were no significant differences between these two treatment conditions.

These findings highlight the potential for psychological interventions as valuable components of cardiac rehabilitation programs. Addressing psychological issues such as stress, anxiety, and depression improves adherence to rehabilitation regimens, enhances quality of life, and reduces cardiovascular risks [26]. Studies have shown that programs integrating tailored psychological interventions, including stress management, psychoeducation, and cognitive–behavioral therapy, offer measurable benefits in various clinical trials, such as improvements in emotional well-being, physical activity levels, and overall cardiovascular health [27,28]. These insights underscore the importance of addressing mental health as a component contributing to the effectiveness of cardiac rehabilitation.

Given the similar effectiveness of both therapies, it is worth considering which one may be the better solution. Therefore, cost-effectiveness is an aspect worth considering. NSDR relaxation, which primarily relies on therapeutic recordings, has lower initial and ongoing costs compared to VR therapy. The implementation of NSDR relaxation does not require expensive equipment or extensive training, making it a more cost-effective option for many healthcare providers. This could be particularly beneficial in settings with limited resources or in outpatient rehabilitation programs where cost constraints are a significant consideration. Additionally, the simplicity and ease of use of NSDR relaxation could facilitate its implementation in the third phase of CR, which is intended to last for the remainder of a patient’s life. It is important to remember that it is challenging to ensure a patient’s participation in therapy over such an extended period. Therefore, therapeutic recordings that the patient can use anywhere and anytime may be an interesting and effective solution. Additionally, this can be carried out at home, which greatly reduces the cost of psychological care for a cardiac patient. All the patient needs is a mobile phone to which the therapeutic recording will be sent.

Another factor to consider is the adaptability of these therapies to different patient needs and preferences. The side effects of short-term VR application are generally rare and harmless [29]. However, when a patient remains in VR for more than 20 min, the risk of VR sickness (including dizziness, headaches, nausea, and eye strain) can increase [30]. The environment and course of VR therapy have been designed to be safe for patients, and no side effects related to VR sickness have been noted. However, in our previous study using VR therapy, we observed a 44% drop-out rate in the experimental group [15]. Patients primarily withdrew from the study due to vision problems and concerns about the impact of VR on pacemaker functioning. In contrast, NSDR poses fewer challenges and may be more accessible to a broader patient population. This ease of use could result in higher adherence rates and greater overall acceptance among patients with varying medical conditions and concerns about using technology.

The comparative analysis between NSDR relaxation, VR therapy, and SAT also highlighted a significant limitation of SAT. Despite its widespread use, SAT was found to be insufficient in effectively improving the mental state of cardiac patients. This finding underscores the need for more innovative and tailored psychotherapeutic interventions within CR programs. It is essential to recognize that while SAT has been beneficial in various settings [31], it may not address the complex, multifaceted psychological needs of cardiac patients who often experience a high burden of comorbid mental health issues. Given that living conditions, needs, and risk factors have evolved over the years, therapies like SAT, which were developed in the past, may no longer be sufficiently effective today.

### Limitations and Future Research Directions

While the results are promising, several limitations should be considered. The pilot nature of the study means that the sample size was relatively small, potentially affecting the generalizability of the findings. Future research should aim to replicate these results with larger, more diverse populations to confirm the efficacy of NSDR in different clinical settings. Additionally, the study population primarily consisted of CAD patients participating in the second phase of CR. It would be valuable to explore the effectiveness of this intervention across different stages of rehabilitation, various stages and severities of disease, and in various cardiac conditions. This approach will make it possible to identify patient characteristics that may impact the effectiveness of these therapies, enabling interventions to be more precisely tailored to individual needs in cardiac rehabilitation.

Another limitation is the short duration of the intervention. The study primarily focused on short-term psychological outcomes, and it is crucial to investigate the long-term effects of NSDR relaxation and VR therapy on mental health. Long-term follow-up studies are necessary to determine the sustained impact of these interventions, including their potential to reduce the recurrence of cardiovascular events, improve adherence to rehabilitation programs, and enhance overall patient satisfaction.

In light of our considerations, a personalized approach to therapy selection may be the most effective strategy. Healthcare providers should assess individual patient characteristics, preferences, and potential contraindications to determine the most suitable therapy. Future research should explore the long-term effects and cost–benefit ratio of both NSDR and VR therapy in CAD patient populations. Additionally, studies could investigate the integration of these therapies with traditional cardiac rehabilitation methods to enhance overall patient outcomes. Another important aspect is understanding the mechanisms of action of NSDR relaxation and VR therapy. Examining heart rate variability can provide insights into how these therapies affect the autonomic nervous system. Future research should also investigate the potential correlations between psychological outcomes and direct measures of cardiac rehabilitation effectiveness, such as exercise capacity, cardiovascular health markers, and long-term survival rates. Additionally, given that VR therapy is stimulating while NDSR relaxation is calming, neuroimaging methods could provide valuable insights into the mechanisms and effectiveness of these therapeutic approaches.

## 5. Conclusions

Despite its limitations, this study provided preliminary evidence supporting the use of NSDR as an effective psychotherapeutic intervention in the context of CR. Both NDSR relaxation and VR therapy, with similar effectiveness, offer promising alternatives to traditional methods, addressing the need for more effective psychological support in patients with CAD. Integrating such innovative techniques into comprehensive CR programs can enhance patient outcomes, improve adherence to rehabilitation protocols, and ultimately contribute to better long-term health. Given the potential benefits of addressing psychological health within cardiac rehabilitation, it is likely that such interventions could not only enhance emotional resilience but also contribute to the overall effectiveness and long-term success of recovery programs. Future research should continue to explore and refine these interventions, ensuring their optimal integration into clinical practice. By expanding our understanding of the mechanisms underlying their effectiveness and identifying the most appropriate contexts for their use, we can enhance the overall care and recovery of cardiac patients.

## Figures and Tables

**Table 1:** Baseline characteristics of participants.

Variable	NSDR Group	VR Group	CON Group	*p* Value
*N*	15	15	15	-
*n* (%) of women	7 (46.67)	7 (46.67)	7 (46.67)	0.97 ^b^
Age, years	61.07 (11.55)	65.40 (9.76)	64.13 (7.80)	0.48 ^a^
Body mass, kg	77.79 (14.02)	75.73 (14.96)	82.33 (13.94)	0.44 ^a^
Body height, cm	169.64 (8.85)	165.80 (9.73)	168.00 (10.11)	0.56 ^a^
Body mass index, kg/m^2^	27.10 (4.98)	27.32 (3.28)	28.95 (2.39)	0.34 ^a^
Normal (BMI 18.5–24.9), *n* (%)	5 (35.71)	4 (26.67)	0 (0.00)	0.18 ^b^
Overweight (BMI 25–29.9), *n* (%)	6 (42.86)	7 (46.66)	10 (66.67)	
Obese (BMI > 30), *n* (%)	3 (21.43)	4 (26.67)	5 (33.33)	
Marital status, *n* (%)				
Married	8 (57.14)	11 (73.34)	11 (73.33)	0.26 ^b^
Single	4 (28.57)	4 (26.66)	1 (6.67)	
Widowed	2 (14.29)	0 (0.00)	3 (20.00)	
Education, *n* (%)				
Primary/vocational	4 (28.57)	4 (26.67)	5 (33.33)	0.35 ^b^
Secondary	8 (57.14)	2 (13.33)	6 (40.00)	
Higher	2 (14.29)	9 (60.00)	4 (26.67)	
Employment status, *n* (%)				
Employed	9 (54.29)	4 (26.66)	8 (53.33)	0.32 ^b^
Disability pension	0 (0.00)	1 (6.67)	6 (40.00)	
Retired	5 (35.71)	10 (66.67)	1 (6.67)	

Continuous variables are expressed as mean (standard deviation). Abbreviations: NSDR group: non-sleep deep rest relaxation group; VR group: virtual reality therapy group; CON group: control group; BMI: body mass index; ^a^: analysis of variance; ^b^: chi-square test.

**Table 2:** Mean values (SD) of measured outcomes.

Outcome	Measurement Time Point	Groups	Mean (SD)	*p* Value *
HADS	Before intervention	NSDR group	12.86 (7.12)	0.94
		VR group	12.47 (7.95)	
		CON group	13.27 (3.35)	
	After intervention	NSDR group	8.93 (5.08)	0.09
		VR group	11.2 (6.86)	
		CON group	13.53 (3.96)	
HADS-A	Before intervention	NSDR group	7.14 (4.11)	0.92
		VR group	6.60 (4.14)	
		CON group	6.73 (2.25)	
	After intervention	NSDR group	5.14 (2.83)	0.17
		VR group	6.53 (3.56)	
		CON group	7.20 (2.24)	
HADS-D	Before intervention	NSDR group	5.71 (3.87)	0.81
		VR group	5.87 (4.07)	
		CON group	6.53 (2.9)	
	After intervention	NSDR group	3.79 (2.72)	0.11
		VR group	4.67 (3.77)	
		CON group	6.33 (3.09)	
PSQ	Before intervention	NSDR group	54.79 (16.07)	0.74
		VR group	56.07 (19.93)	
		CON group	59.60 (15.15)	
	After intervention	NSDR group	49.50 (15.05)	0.02
		VR group	48.60 (19.38)	
		CON group	63.73 (13.18)	
ES	Before intervention	NSDR group	16.79 (6.09)	0.83
		VR group	17.00 (6.21)	
		CON group	18.00 (5.09)	
	After intervention	NSDR group	16.43 (5.52)	0.12
		VR group	14.73 (5.41)	
		CON group	18.67 (4.32)	
IS	Before intervention	NSDR group	17.79 (5.75)	0.97
		VR group	17.93 (8.05)	
		CON group	18.40 (5.38)	
	After intervention	NSDR group	16.57 (5.71)	0.22
		VR group	15.93 (7.65)	
		CON group	19.67 (4.52)	
ET	Before intervention	NSDR group	20.21 (5.49)	0.45
		VR group	21.13 (7.45)	
		CON group	23.20 (6.36)	
	After intervention	NSDR group	16.50 (6.15)	<0.01
		VR group	17.93 (8.09)	
		CON group	25.40 (6.07)	

Abbreviations: HADS: hospital anxiety (A) and depression (D) scale; PSQ: perception of stress questionnaire; ES: external stress; IS: intrapsychic stress; ET: emotional tension; NSDR group: non-sleep deep rest relaxation group; VR group: virtual reality therapy group; CON group: control group; *: analysis of variance.

**Table 3:** ANOVA results (time × group).

Outcome	Mean Square	*F*	η_p_^2^	*p* Value
HADS	32.46	5.51	0.21	0.01
HADS-A	12.08	4.19	0.17	0.02
HADS-D	5.48	2.74	0.12	0.08
PSQ	283.56	7.27	0.26	<0.01
ES	16.60	2.26	0.10	0.12
IS	21.72	3.57	0.15	0.04
ET	79.33	8.77	0.30	<0.001

Abbreviations: HADS: hospital anxiety (A) and depression (D) scale; PSQ: perception of stress questionnaire; ES: external stress; IS: intrapsychic stress; ET: emotional tension.

**Table 4:** Pairwise comparison of changes in measured outcomes.

Outcome	Groups	Mean Difference (95% CI)	*p* Value *
HADS	NSDR group–CON group	−4.20 (−7.30 to −1.09)	<0.01
	VR group–CON group	−1.53 (−4.58 to 1.51)	0.68
	NSDR group–VR group	−2.66 (−5.76 to 0.44)	0.13
HADS-A	NSDR group–CON group	−2.47 (−4.64 to −0.30)	0.03
	VR group–CON group	−0.53 (−2.67 to 1.60)	1.00
	NSDR group–VR group	−1.93 (−4.10 to 0.24)	0.11
HADS-D	NSDR group–CON group	−1.73 (−3.53 to 0.08)	0.08
	VR group–CON group	−1.00 (−2.77 to 0.77)	0.53
	NSDR group–VR group	−0.73 (−2.53 to 1.08)	0.99
PSQ	NSDR group–CON group	−9.42 (−17.40 to −1.44)	0.02
	VR group–CON group	−11.60 (−19.44 to −3.76)	<0.01
	NSDR group–VR group	2.18 (−5.80 to 10.16)	1.00
ES	NSDR group–CON group	−1.02 (−4.49 to 2.44)	1.00
	VR group–CON group	−2.93 (−6.33 to 0.47)	0.13
	NSDR group–VR group	1.91 (−1.55 to 5.37)	0.56
IS	NSDR group–CON group	−2.48 (−7.35 to 2.39)	0.67
	VR group–CON group	−2.33 (−7.12 to 2.45)	0.73
	NSDR group–VR group	−0.15 (−5.02 to 4.72)	1.00
ET	NSDR group–CON group	−5.91 (−9.76 to −2.07)	<0.01
	VR group–CON group	−5.40 (−9.18 to −1.62)	<0.01
	NSDR group–VR group	−0.51 (−4.36 to 3.33)	1.00

Abbreviations: HADS: hospital anxiety (A) and depression (D) scale; PSQ: perception of stress questionnaire; ES: external stress; IS: intrapsychic stress; ET: emotional tension; NSDR group: non-sleep deep rest relaxation group; VR group: virtual reality therapy group; CON group: control group; *: post hoc analysis, using Bonferroni method.

## Data Availability

Data are available on reasonable request to the corresponding author.
